# Impact of α-Targeted Radiation Therapy on Gene Expression in a Pre-Clinical Model for Disseminated Peritoneal Disease when Combined with Paclitaxel

**DOI:** 10.1371/journal.pone.0108511

**Published:** 2014-09-30

**Authors:** Kwon Joong Yong, Diane E. Milenic, Kwamena E. Baidoo, Martin W. Brechbiel

**Affiliations:** Radioimmune & Inorganic Chemistry Section, Radiation Oncology Branch, National Cancer Institute, National Institutes of Health, Bethesda, Maryland, United States of America; University of South Florida, United States of America

## Abstract

To better understand the molecular basis of the enhanced cell killing effected by the combined modality of paclitaxel and ^212^Pb-trastuzumab (Pac/^212^Pb-trastuzumab), gene expression in LS-174T i.p. xenografts was investigated 24 h after treatment. Employing a real time quantitative PCR array (qRT-PCR array), 84 DNA damage response genes were quantified. Differentially expressed genes following therapy with Pac/^212^Pb-trastuzumab included those involved in apoptosis (*BRCA1, CIDEA, GADD45α, GADD45γ, GML, IP6K3, PCBP4, PPP1R15A, RAD21*, and *p73*), cell cycle (*BRCA1, CHK1, CHK2, GADD45α, GML, GTSE1, NBN, PCBP4, PPP1R15A, RAD9A*, and *SESN1*), and damaged DNA repair (*ATRX, BTG2, EXO1, FEN1, IGHMBP2, OGG1, MSH2, MUTYH, NBN, PRKDC, RAD21*, and *p73*). This report demonstrates that the increased stressful growth arrest conditions induced by the Pac/^212^Pb-trastuzumab treatment suppresses cell proliferation through the regulation of genes which are involved in apoptosis and damaged DNA repair including single and double strand DNA breaks. Furthermore, the study demonstrates that ^212^Pb-trastuzumab potentiation of cell killing efficacy results from the perturbation of genes related to the mitotic spindle checkpoint and BASC (BRCA1-associated genome surveillance complex), suggesting cross-talk between DNA damage repair and the spindle damage response.

## Introduction

Microtubule-targeting cancer therapies, such as paclitaxel (Pac), perturb the dynamics of the mitotic spindle, blocking cells in mitosis from progressing through the cell cycle by activating the mitotic checkpoint. Paclitaxel is an FDA approved chemotherapeutic treatment for ovarian, breast, and lung carcinomas [1], [2]. This chemotherapeutic has been combined with low LET photon radiation for the treatment of cancer. High LET radiation such as α-particles, however, is more effective in cell killing than low LET radiation [3]–[5]. To take advantage of the cell killing efficiency of α-radiation, α-emitting radionuclides have been successfully carried on vector antibodies in radioimmunotherapy (RIT) to effect targeted therapy of cancer [6]–[9]. In combination with chemotherapeutics, such as gemcitabine and paclitaxel, highly enhanced therapeutic efficacy has been demonstrated by the α-particle targeted radioimmunotherapeutic ^212^Pb-trastuzumab [10], [11]. Application of α-emitter immunoconjugates, such as ^212^Pb-trastuzumab either as monotherapy or in combination with chemotherapeutics, are promising therapeutic options for treatment of carcinomas that are characterized by dissemination of single tumor cells in the peritoneum like ovarian or gastric cancer. This success has translated to an ongoing Phase I human clinical trial at the University of Alabama with 16 patients treated to date [12].

Exposure of cells to ionizing radiation activates multiple signal transduction pathways resulting in complex alterations in gene expression. Despite a long history of studies regarding the mechanisms of action of paclitaxel and photon radiation used individually, little is understood about how paclitaxel and radiation together promote *in vivo* tumor cell death. In particular, gene modulations associated with the cytocidal response have not been clearly defined following exposure of cells to α-particle RIT combined with paclitaxel [13], [14]. A recent report from this laboratory showed that paclitaxel potentiates ^212^Pb-trastuzumab cytotoxicity, in part, by perturbing the mitotic spindle checkpoint [15]. Gene expression profiling provides a potentially powerful approach towards understanding the molecular basis of the cellular response to therapeutic agents. The use of high LET radiation such as α-particles originating from radionuclides such as ^211^At and ^213^Bi on different biological systems has identified gene expression profiles [16], [17]. Irradiation results in major damage to DNA while paclitaxel affects microtubules. Modifications in gene expression invoked by Pac/^212^Pb-trastuzumab may thus derive mainly from perturbation of the microtubule network and DNA damage signaling pathways. In order to better understand the molecular basis of the therapeutic efficacy of targeted α-radiation in combination with paclitaxel, changes in gene expression induced by Pac/^212^Pb-trastuzumab therapy were investigated. For this purpose, mice bearing human colon cancer LS-174T i.p. xenografts were pre-treated with paclitaxel, followed 24 h later by treatment with ^212^Pb-trastuzumab. The gene expression of LS-174T i.p. tumor xenografts from mice that received paclitaxel plus specifically targeted α-RIT (^212^Pb-trastuzumab) was compared to paclitaxel plus a non-specifically labeled control (^212^Pb-HuIgG), paclitaxel alone, and untreated control tumors. Gene expression was quantified using a real time quantitative PCR (qRT-PCR) array covering 84 genes in the DNA damage signaling pathway.

## Materials and Methods

### Cell line

The human colon carcinoma cell line (LS-174T) was used for all *in vivo* studies. LS-174T was grown in supplemented Dubelcco's Modified Eagle's Medium (DMEM) as previously described in the published reference [18]. All media and supplements were obtained from Lonza (Walkersville, MD). The cell line has been screened for mycoplasma and other pathogens before *in vivo* use according to National Cancer Institute (NCI) Laboratory Animal Sciences Program policy. No authentication of the cell line was conducted by the authors.

### Chelate synthesis, mAb conjugation, and radiolabeling

The synthesis, characterization, and purification of the bifunctional ligand TCMC has been previously described [19]. Trastuzumab (Genentech, South San Francisco, CA) was conjugated with TCMC by established methods using a 10-fold molar excess of ligand to mAb. A 10 mCi (0.37 GBq) ^224^Ra/^212^Pb generator was purchased from AlphaMed (Lakewood, NJ). HuIgG was also conjugated with the TCMC ligand and radiolabeled, providing a non-specific control antibody for the experiments.

### Tumor model, treatment, and tumor harvesting

Studies were performed with 19–21 g female athymic mice (NCI-Frederick) bearing intraperitoneal (i.p.) LS-174T xenografts as previously reported [19]. The viability of the LS-174T cells (>95%) was determined using trypan-blue. Athymic mice were injected i.p. with 1×10^8^ LS-174T cells in 1 mL of DMEM. The inoculum size for this cell line represents the minimum amount of cells required for tumor growth in 100% of the mice. Two days after tumor cell inoculation, the mice (n = 10–15) were given i.p. injections of paclitaxel (600 µg; Hospira, Inc, Lake Forest, IL). ^212^Pb-trastuzumab ((10 µCi (0.37 MBq) in 0.5 mL PBS)) was administered i.p. to the mice 24 h later. Mice were euthanized in their home cages with the specialized euthanasia lid attached to the CO_2_ line. The flow rate of CO_2_ was 2 L/min. When breathing ceases for all mice, the mice were removed from the CO_2_-filled cage. After euthanasia, the tumor tissues from the 24 h time point were pooled together, macroscopically inspected, and adherent tissues were removed. The tumor tissues were then thoroughly rinsed in ice-cold PBS 3 times, divided, and processed accordingly for each assay. This treatment group was compared with sets of tumor bearing mice (n = 10–15) that received paclitaxel or Pac/^212^Pb-HuIgG. All animal protocols were approved by the NCI Animal Care and Use Committee.

### RNA purification

Total RNA was isolated from harvested tumor tissues using the RNeasy Mini Kit (Qiagen, Santa Clarita, CA) according to the manufacturer's instruction and stored at −80°C until assayed. Purity of isolated total RNA was measured using a NanoDrop spectrophotometer (Thermo Scientific, Wilmington, DE) and PCR with β-actin primers. Only total RNA with A260/A280 ratio >1.9 and without detectable contamination of DNA (PCR) were employed for gene expression array (qRT-PCR array).

### Human DNA damage PCR array

The cDNA were reverse transcribed from RNA using the First Strand cDNA Synthesis Kit (SABiosciences, Frederick, MD). Comparison of the relative expression of the 84 DNA damage related genes was characterized by human DNA Damage PCR array (SABiosciences) and the RT^2^ real-time SYBR Green/Rox PCR master mix (SABiosciences) on a 7500 real time PCR system (Applied Biosystems, Rockville, MD). The array includes genes involved in apoptosis, cell cycle and damaged DNA binding and repair (Table S1). Data was analyzed using the RT^2^ Profiler PCR Array Data Analysis v3.5 software (Qiagen). The fold change in gene expression was calculated using the equation 2(−ΔΔC_T_). In cases in which a gene was down-regulated (less than 1 fold change), the value was reported as the negative inverse.

### Chromatin immunoprecipitation

The chromatin immunoprecipitation (ChIP) assay kit (Upstate Biotechnology, Billerica, MA) was utilized according to the manufacturer's instructions with minor adjustments. The lysates were prepared and aliquoted. Ten µL of antibody (1∶100) for E2F1 (Upstate Biotechnology) was added to a lysate aliquot; the resulting DNA-protein complexes were isolated by protein G agarose beads. The samples were subjected to 65°C for 5 h, the DNA extracted, and dissolved in elution reagent. The immunoprecipitated DNA was amplified by PCR using *BRCA1* and *p73* promoter specific primers (Applied Biosystems) and analyzed by electrophoresis using 2% agarose gels.

### Immunblot analysis

Immunoblot analysis following standard procedures was performed with total protein isolates using tissue protein extraction reagent (Thermo Scientific, Asheville, NC) containing protease inhibitors (Roche, Indianapolis, IN). Fifty µg of total protein per lane was separated on a 4–20% tris-glycine gel and transferred to a nitrocellulose membrane. Antibodies against MAD2 (Cell Signaling, Beverly, MA), CYCLIN B1, EMI1, and GEMININ (SantaCruz, Santa Cruze, CA) were used at a dilution of 1∶1000 in PBS containing 5% BSA and 0.05% Tween-20. Horseradish peroxidase conjugated rabbit secondary antibodies were used at 1∶5000 in 3% non-fat dry milk. The blots were developed using the ECL plus chemoluminescent detection kit (GE Healthcare, Pascataway, NJ and images were acquired using a Fuji LAS 4000 imager (GE Healthcare, Pascataway, NJ).

### Statistics

A minimum of at least three independent experiments were conducted for each treatment described. Student *t* test was used for paired data and multiple comparisons were performed with the ANOVA. A *p*-value <0.05 was considered statistically significant.

## Results

To explore the molecular basis of α-particle RIT in combination with paclitaxel therapy in LS-174T i.p. xenografts, qRT-PCR array was employed for gene expression analysis of 84 genes of the DNA damage signaling pathway in three independent experiments. The qRT-PCR array identified the genes significantly up- or down-regulated at 24 h after radioactivity injection. Each treatment group was separately compared with the untreated group using a 2-fold change cut-off.

### Pac/^212^Pb-trastuzumab-induced cell killing is associated with an increased expression of genes involved in apoptosis

Of the 84 genes examined (Table S1), 13 genes are involved in regulation of the apoptotic process and eight of those genes (*CIDEA, GADD45α, GADD45γ, GML, IP6K3, PCBP4, PPP1R15A*, and *p73*) were up-regulated in tumors collected from mice treated with Pac/^212^Pb-trastuzumab (Table 1). Of these up-regulated genes, the greatest impact was on the expression of *CIDEA* (14.0-fold increase, *p*<0.0067), *GML* (26.4-fold increase, *p*<0.0004), and *IP6K3* (18.1-fold increase, *p*<0.0001) in the LS-174T tumor xenografts. There was also a clear difference between this group and the groups that received the Pac/^212^Pb-HuIgG or only Pac (Pac/^212^Pb-trastuzumab *vs* paclitaxel, *p*<0.01; Pac/^212^Pb-trastuzumab *vs* Pac/^212^Pb-HuIgG, *p*<0.01). Among the other genes for which an up-regulation of expression was noted, the differences between the Pac/^212^Pb-trastuzumab and Pac/^212^Pb-HuIgG were either modest (GADD45α), or negligible (GADD45γ and PCBP4). Treatment with either Pac/^212^Pb-trastuzumab or Pac/^212^Pb-HuIgG resulted in an increase in the expression of *p73*, however, a greater fold increase was observed in the latter group. The expression of two genes, *BRCA1* and *Rad21*, were down-regulated by Pac/^212^Pb-trastuzumab, albeit their expression was also found to be down-regulated to a lesser degree by Pac/^212^Pb-HuIgG.

**Table 1:** Expression of genes involved in apoptosis in LS-174T i.p. xenografts following treatment with Paclitaxel and ^212^Pb-trastuzumab.

Symbol	Gene name	GeneBank ID	Fold change					
			Paclitaxel-^212^Pb-trastuzumab	*p*	Paclitaxel-^212^Pb-HuIgG	*p*	Paclitaxel	*p*
BRCA1	Breast Cancer 1, early onset	NM_007294	-3.8	0.0005	−3.0	0.0022	−2.1	0.0061
CIDEA	Cell death-inducing DEFA-like effector a	NM_001279	14.0	0.0067	3.8	0.0654	1.1	0.4741
GADD45α	Growth arrest and DNA-damage-inducible, alpha	NM_001924	5.9	0.0001	3.8	0.0002	1.9	0.0962
GADD45γ	Growth arrest and DNA-damage-inducible, gamma	NM_006705	10.9	0.0001	9.4	0.0002	2.4	0.0067
GML	Glycosylphosphatidylinositol anchored molecule like protein	NM_002066	26.4	0.0004	2.7	0.0023	−1.4	0.4430
IP6K3	Inositol hexakisphosphate kinase 3	NM_054111	18.1	0.0001	8.7	0.0843	−1.1	0.6288
PCBP4	Poly(rC)binding protein 2	NM_020418	3.1	0.0116	2.8	0.0012	1.6	0.0205
PPP1R15A	Protein phosphatase 1, regulatory subunit 15A	NM_014330	2.6	0.0115	1.4	0.0399	−1.1	0.4490
RAD21	RAd21 homolog	NM_006265	−3.1	0.0001	−2.5	0.0002	−1.7	0.0184
p73	Tumor protein p73	NM_005427	2.6	0.0009	5.6	0.0021	2.9	0.0628

Mice bearing i.p. LS-174T xenografts were treated by Pac/^212^Pb-trastuzumab for 24 h. qRT-PCR array was used for gene expression analysis in three independent experiments. The numbers indicate fold change compared to untreated control (2-fold change cut-off). Additional groups included paclitaxel alone and Pac/^212^Pb-HuIgG as a nonspecific control antibody. Results represent the average of a minimum of three replicates. A *p*-value <0.05 was considered significantly significant.

### Pac/^212^Pb-trastuzumab-induced cell killing may be associated with differential expression of genes involved in the regulation of cell cycle arrest and cell cycle check point

Genes involved in regulation of cell cycle arrest (15 genes) and cell cycle checkpoint (8 genes) were another component of the array used in this gene profiling study. Expression of six genes was found to be up-regulated while five genes were down-regulated 24 h after the sequential exposure to Pac and ^212^Pb-trastuzumab (Table 2). The genes associated with cell cycle arrest that demonstrated an increase in expression were *GADD45α, GML, PCBP4, PP1R15A, RAD9A*, and *SESN1*. In contrast, the *CHK1, CHK2* and *GTSE1* genes were down-regulated. When compared to the group treated with Pac/^212^Pb-HuIgG, the Pac/^212^Pb-trastuzumab treatment elicited a modestly greater response of *CHK1, RAD9A*, and *SENS1*. The Pac/^212^Pb-trastuzumab treatment had the greatest effect on the expression of *GML* (26.4-fold increase, *p*<0.0004), compared to the set of tumors treated with Pac and the non-specifically targeted ^212^Pb-HuIgG (Pac/^212^Pb-trastuzumab *vs* paclitaxel, *p*<0.01; Pac/^212^Pb-trastuzumab *vs* Pac/^212^Pb-HuIgG, *p*<0.01). *GML* has been suggested to have a possible role in arresting cell cycle in the G2/M phase [20]. Of the eight genes involved in the cell cycle checkpoint, an alteration in only *BRCA1* and *NBN* gene expression was noted. For *NBN* genes, the difference between the Pac/^212^Pb-trastuzumab and Pac/^212^Pb-HuIgG treated tumor tissue was negligible. There was approximately a four-fold reduction in *BRCA1* expression for Pac/^212^Pb-trastuzumab versus a three-fold reduction of expression for Pac/^212^Pb-HuIgG treatment as compared to a two-fold reduction for paclitaxel treatment alone (Pac/^212^Pb-trastuzumab *vs* paclitaxel, *p*<0.05).

**Table 2:** Expression of genes involved in cell cycle in LS-174T i.p. xenografts following treatment with Paclitaxel and ^212^Pb-trastuzumab.

Symbol	Gene name	GeneBank ID	Fold change					
			Paclitaxel-^212^Pb-trastuzumab	*p*	Paclitaxel-^212^Pb-HuIgG	*p*	Paclitaxel	*p*
BRCA1	Breast Cancer 1, early onset	NM_007294	−3.8	0.0005	−3.0	0.0022	−2.1	0.0061
CHK1	CHK1 checkpoint homolog	NM_001274	−4.0	0.0001	−3.2	0.0002	−2.4	0.0018
CHK2	CHK2 checkpoint homolog	NM_007194	−2.0	0.0016	−1.9	0.0025	−2.4	0.0032
GADD45α	Growth arrest and DNA-damage-inducible, alpha	NM_001924	5.9	0.0001	3.8	0.0002	1.9	0.0962
GML	Glycosylphosphatidylinositol anchored molecule like protein	NM_002066	26.4	0.0004	2.7	0.0023	−1.4	0.4430
GTSE1	G-2 and S-phase expressed 1	NM_016426	−9.3	0.0014	−4.3	0.0025	−3.1	0.0043
NBN	Nibrin	NM_002485	−2.0	0.0303	−2.1	0.0065	−1.4	0.0999
PCBP4	Poly(rC)binding protein 2	NM_020418	3.1	0.0116	2.8	0.0012	1.6	0.0205
PPP1R15A	Protein phosphatase 1, regulatory subunit 15A	NM_014330	2.6	0.0115	1.4	0.0399	−1.1	0.4490
RAD9A	RAD9 homolog A	NM_004584	2.4	0.0016	1.6	0.0006	−1.3	0.0132
SESN1	Sestrin1	NM_014454	4.6	0.0001	3.8	0.0006	2.8	0.0004

### Impact of Pac/^212^Pb-trastuzumab on expression of genes involved in damaged DNA repair

The LS-174T tumor xenografts were also probed to identify those genes involved in DNA repair that were affected by the combined treatment modality. These genes fall into several categories (Table S1) which include damaged DNA binding (DDBR; 26 genes) as well as those that comprise single strand break repair, nucleotide excision repair (NER; 12 genes), base-excision (BER; 7 genes) and mismatch repair (MMR; 14 genes). Nine genes pivotal in double-strand break repair (DSB) were also screened using the same qRT-PCR array. The array includes 15 other uncategorized genes that are related to DNA repair. Again, using the criteria of a 2-fold difference, several genes presented with a difference in expression at 24 h subsequent to the administration of Pac/^212^Pb-trastuzumab (Table 3). Among these genes, however, only six demonstrated a clear difference between the tumors harvested from mice that had been treated with Pac/^212^Pb-trastuzumab and those that had received the Pac/^212^Pb-HuIgG. These genes were *ATRX, BTG2, IGHMBP2, FEN1* and *p73*, and *XPC*. The first three, *ATRX, BTG2*, and *IGHMBP2*, fall into the category of other genes related to DNA repair. *FEN1* and *XPC* are involved in repair of DDBR while *p73* is involved in MMR. One caveat to observations related to the latter gene is that the tumors from the Pac/^212^Pb-HuIgG group demonstrated a greater increase (Pac/^212^Pb-trastuzumab *vs* Pac/^212^Pb-HuIgG, *p*<0.05) than the tumors treated with Pac/^212^Pb-trastuzumab (a 2.7- fold increase, *p*<0.0009 *vs* a 5.6-fold increase, *p*<0.0021). *FEN1* was the only gene demonstrating a decrease (a 4.5-fold decrease, *p*<0.0024) in expression (Pac/^212^Pb-trastuzumab *vs* paclitaxel, *p*<0.05; Pac/^212^Pb-trastuzumab *vs* Pac/^212^Pb-HuIgG, *p*<0.05).

**Table 3:** Expression of gene expression involved in DNA repair in LS-174T i.p. xenografts following treatment with Paclitaxel and ^212^Pb-trastuzumab.

Symbol	Gene name	GeneBank ID	Fold change					
			Paclitaxel-^212^Pb-trastuzumab	*p*	Paclitaxel-^212^Pb-HuIgG	*p*	Paclitaxel	*p*
ATRX	Alpha thalassemia/mental retardation	NM_000489	2.8	0.0018	1.4	0.1413	1.1	0.4611
BRCA1	Breast Cancer 1, early onset	NM_007294	−3.8	0.0005	−3.0	0.0022	−2.1	0.0061
BTG2	BTG family, member 2	NM_006763	7.5	0.0006	5.4	0.0003	2.7	0.0001
EXO1	Exonuclease 1	NM_130398	−4.5	0.0004	−4.0	0.0003	−2.1	0.0030
FEN1	Flap structure-specific endonuclease 1	NM_004111	−4.5	0.0024	−5.8	0.0017	−2.4	0.0072
IGHMBP2	Immunoglobulin mu binding protein 2	NM_002180	2.4	0.0013	1.8	0.0307	−1.4	0.0430
MSH2	MutS homolog 2	NM_000251	−2.9	0.0080	−2.7	0.0085	−1.9	0.0249
MUTYH	MutY homolog	NM_012222	2.8	0.0002	2.0	0.0019	−1.4	0.0837
NBN	Nibrin	NM_002485	−2.0	0.0303	−2.1	0.0065	−1.4	0.0999
OGG1	8-oxoguanine DNA glycosylase	NM_002542	−2.2	0.1217	−2.2	0.1214	−1.9	0.2046
PNKP	Polynucleotise kinase 3′-phosphatase	NM_007254	2.4	0.0063	2.1	0.0014	−1.5	0.0084
PPP1R15A	Protein phosphatase 1, regulatory unit 15A	NM_014330	2.6	0.0115	1.4	0.0399	−1.1	0.4490
PRKDC	Protein kinase, DNA-activated, catalytic polypeptide	NM_006904	−2.7	0.0187	−2.5	0.0126	−1.9	0.0306
RAD18	RAD18 homolog	NM_020165	−2.3	0.0030	−1.9	0.0003	−1.6	0.0001
RAD21	RAD21 homolog	NM_006265	−3.1	0.0001	−2.5	0.0002	−1.7	0.0184
SEMA4A	Semadomain, immunoglobulin domain, cycloplastic domain 4A	NM_022367	2.8	0.0350	2.7	0.0031	1.3	0.3131
p73	Tumor protein p73	NM_005427	2.7	0.0009	5.6	0.0021	2.9	0.0628
XPC	Xeroderma pigmentosum, complementation group C	NM_004628	5.1	0.0001	3.7	0.0001	1.9	0.0141
XRCC2	X-ray repair complementing defective repair in Chinese hamster cells 2	NM_005431	−3.5	0.0020	−3.0	0.0003	−2.2	0.0025

Of the remaining genes for which differential expression was observed, nine were down-regulated (*BRCA1, EXO1, MSH2, NBN, OGG1, PRKDC, RAD18, RAD21* and *XRCC2*), while only 3 were up-regulated (*MUTYH, PNKP* and *SEM4A*). Eight of these genes play a role in DDBR, three in MMR, two each in NER and DSB repair, and finally one in BER. There were only modest to negligible differences between Pac/^212^Pb-trastuzumab and Pac/^212^Pb-HuIgG treated tumors amongst these genes.

### Pac/^212^Pb-trastuzumab down-regulates gene expression related to BASC

**Figure 1:**
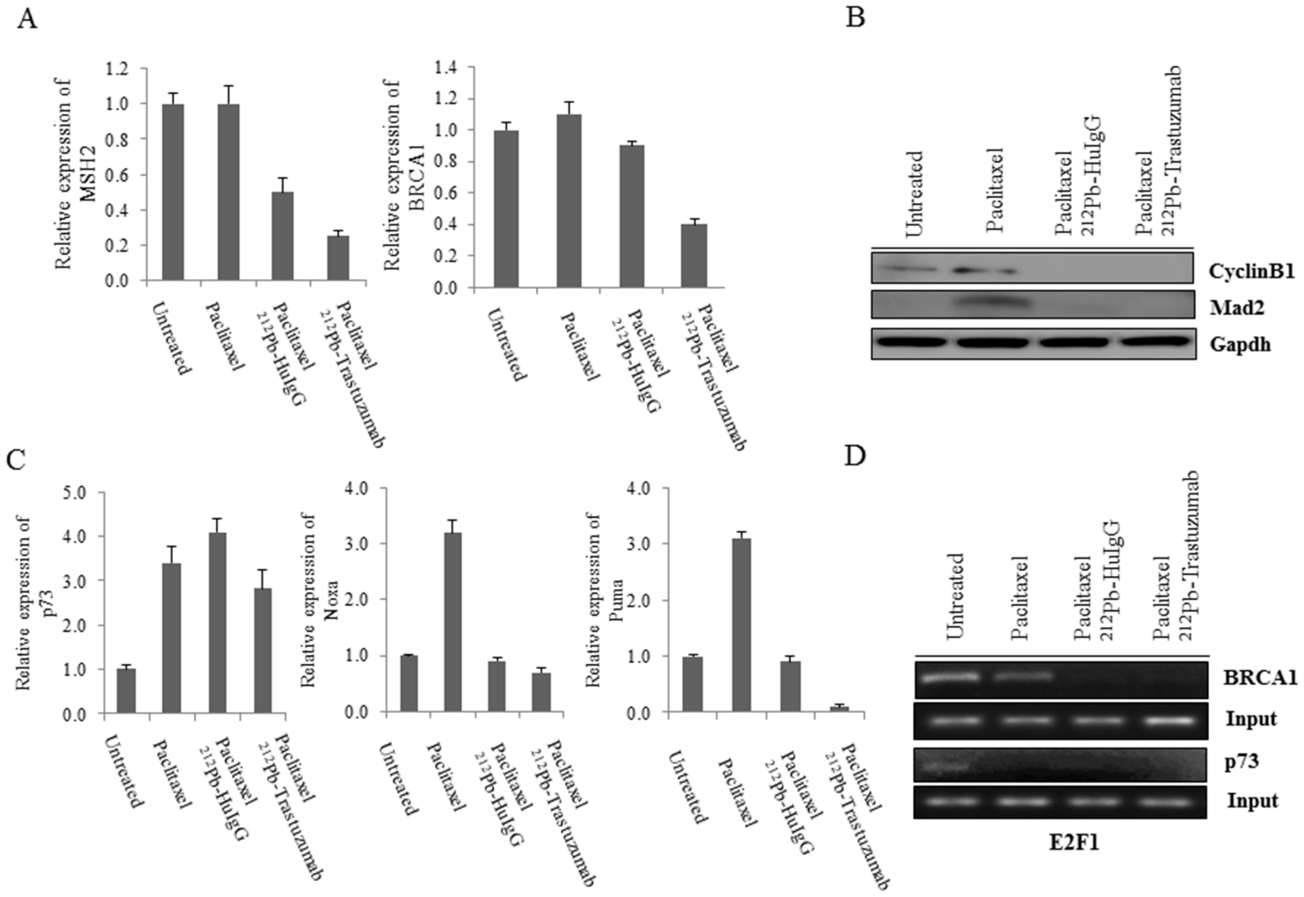
Expression of BASC (BRCA1-associated genome surveillance complex) related genes and *p73* expression in response to Pac/^212^Pb-trastuzumab. Mice bearing i.p. LS-174T xenografts were treated by Pac/^212^Pb- trastuzumab for 24 h. A. Expression of *MSH2* and *BRCA1* was determined by RT-PCR. Results represent the average of a minimum of three replications. B. Immunoblot analysis for MAD2 and CYCLIN B1 was performed with tumor collected 24 h after Pac/^212^Pb-trastuzumab treatment. MAD2 and CYCLIN B1 were detected 22 kDa and 48 kDa, respectively. Equal protein loading control was GAPDH. C. Expression of *p73, NOXA*, and *PUMA* was determined by RT-PCR. Results represent the average of a minimum of three replications. D. Binding abundance to E2F1 was determined by ChIP using specific primers for *p73 and BRCA1*.

BASC (BRCA1-associated genome surveillance complex) is a multi-subunit complex, which includes BRCA1 and other DNA damage proteins such as MSH2-MSH6 and MLH1, as well as ATM, NBS1 (NBN), MRE11 and BLM [21]. BRCA1 may function as a coordinator of multiple activities required for maintenance of genomic integrity during the process of DNA replication. Genetic instability caused by BRCA1 deficiency triggers cellular responses to DNA damage that blocks cell proliferation and induces apoptosis [22]. Among genes identified in the profile, *BRCA1, MSH2*, and *NBN*, which are associated with BASC, were found to be down-regulated after the Pac/^212^Pb-trastuzumab treatment. To investigate the effect on BASC by Pac/^212^Pb-trastuzumab, the expression of *MSH2* and *BRCA1* were determined at the transcriptional level. Indeed, Pac/^212^Pb-trastuzumab reduced expression of *MSH2* and *BRCA1* at the transcriptional level (Untreated *vs* Pac/^212^Pb-trastuzumab, *p*<0.01; Pac/^212^Pb-trastuzumab *vs* Pac/^212^Pb-HuIgG, *p*<0.05), suggesting that transcription-coupled repair (Mismatch repair; *MSH2* and DNA double stand repair; *BRCA1*) might be defective (Figure 1A).

*CHK*-mediated phosphorylation of *BRCA1* is required for proper and timely assembly of mitotic spindles. Down-regulation of *BRCA1* reduces the mitotic index and triggers premature CYCLIN B1 degradation and decreases expression of genes that are involved in the spindle checkpoint including MAD2 [23]. To examine the role of BRCA1 in mitosis, the expression of DNA damage response genes such as CYCLIN B1 and MAD2 were determined using immunoblot analysis (Figure 1B). Pac/^212^Pb-trastuzumab revoked the expression of CYCLIN B1 and MAD2, induced by the administration of paclitaxel alone suggesting that down-regulation in BASC by Pac/^212^Pb-trastuzumab may result in genomic instability. The decrease in the level of these two proteins was also observed in the tumors that had been treated with Pac/^212^Pb-HuIgG.

### Pac/^212^Pb-trastuzumab may induce chromosomal instability as a result of interfere with E2F1/p73 signaling

Reports have indicated that p73 interacts with spindle assembly checkpoint (SAC) proteins and that loss of p73 causes mislocalization at the kinetochore and reduced kinase activity of BubR1, leading to chromosome instability [24]. The expression of *p73* at the transcriptional level appeared to be up-regulated, as the *p73* gene expression was increased for paclitaxel alone, but there is a reduction in *p73* expression with Pac/^212^Pb-trastuzumab (Table 3). To examine the effect on genome instability induced by Pac/^212^Pb-trastuzumab, the expression of *p73* and *NOXA/PUMA*, down-stream effectors of p73, was determined at the transcriptional level. Pac/^212^Pb-trastuzumab treatment seemed to marginally increase the expression of *p73* at the gene level (Figure 1C). However, the expression of *p73* downstream effectors that was apparent after the initial Pac treatment was reduced (Untreated *vs* Pac/^212^Pb-trastuzumab, *p*<0.05). p73 is a transcriptional target of E2F1 and therefore p73 regulation may be mediated by E2F1 [25]. BRCA1 promoter also has E2F binding sites to bring about transcriptional regulation [26]. With these in mind, the abundances of E2F1 association with *BRCA1* and *p73* promoters in tumors exposed to Pac/^212^Pb-trastuzumab were evaluated using the ChIP assay. Pac/^212^Pb-trastuzumab and Pac/^212^Pb-HuIgG (Figure 1D) attenuated the binding capacity at *BRCA1* and *p73* promoters, implicating a disturbance in activation of E2F1/p73 signaling.

### Pac/^212^Pb-trastuzumab may induce chromosomal instability by the regulating mitotic spindle checkpoint

**Figure 2:**
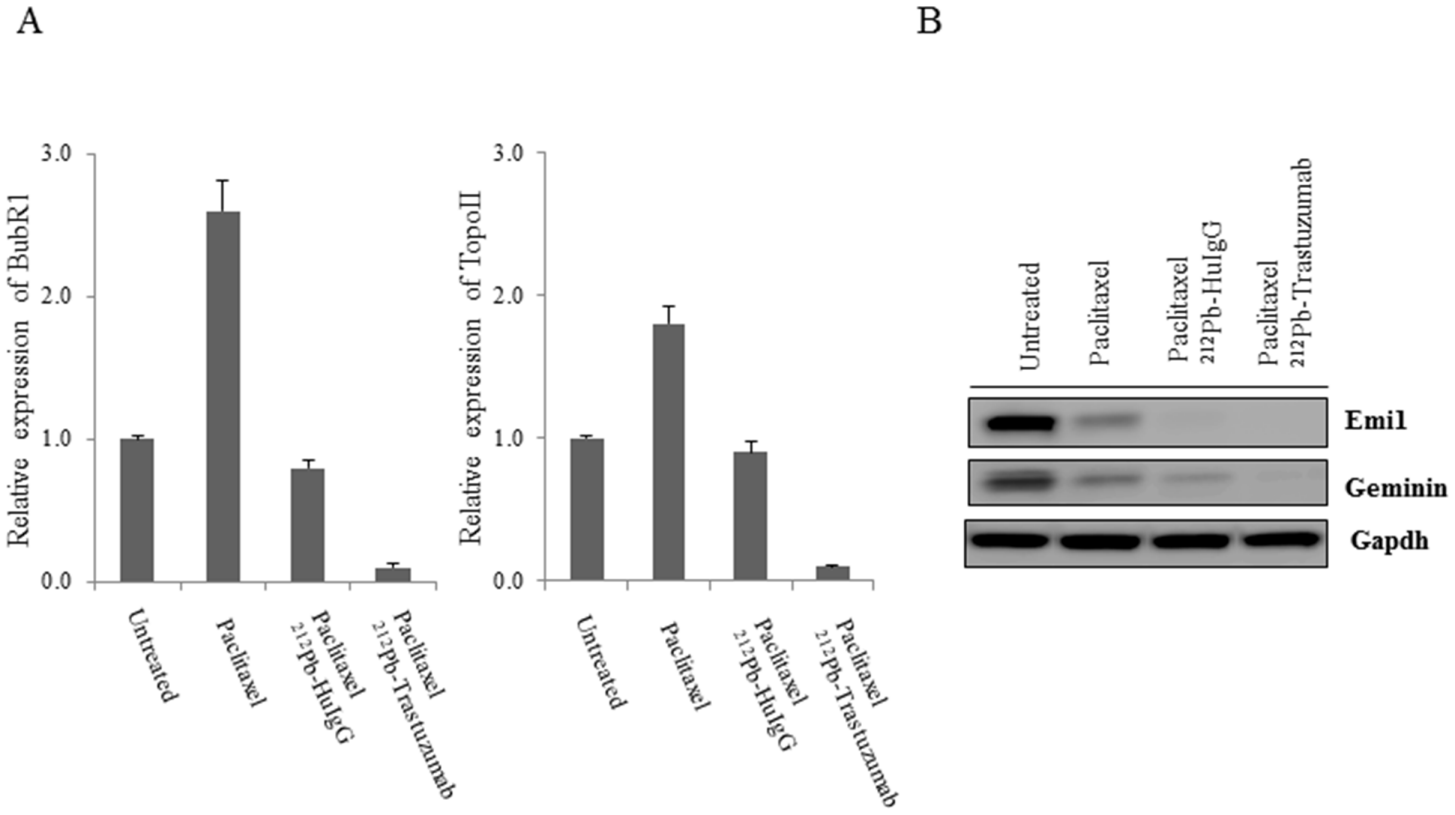
Pac/^212^Pb-trastuzumab may induce chromosomal instability as a result of suppression of BUBR1 and EMI1 expression. Mice bearing i.p. LS-174T xenografts were treated by Pac/^212^Pb-trastuzumab for 24 h. A. Expression of *BUBR1* and *TOPOII* was determined by RT-PCR using specific primers for *BUBR1* and *TOPOISOMERASE II*. Results represent the average of a minimum of three replications. B. Immunoblot analysis for EMI1 and GEMININ was performed with tumor tissue collected 24 h after Pac/^212^Pb- trastuzumab treatment. The EMI1 and GEMININ were detected at 56 kDa and 35 kDa, respectively. Equal protein loading control was GAPDH.

Reduced BUBR1 activity results in the premature activation of the anaphase promoting complex/cyclosome (APC/C), which negatively regulates its substrates such as GEMININ [27]. To further investigate the effect on genome instability induced by Pac/^212^Pb-trastuzumab, the expression of *BUBR1*, one of the spindle assembly checkpoint (SAC) proteins, was determined at the transcriptional level (Figure 2A). Upon incubation with paclitaxel alone, *BUBR1* expression was found to be elevated. The increase in expression of *BUBR1* was reversed at the transcription level upon subsequent treatment with ^212^Pb-trastuzumab consistent with an earlier study from this laboratory [15]. Topoisomerase II helps to bring about a high order of compaction of chromatin to form condensed mitotic chromosomes [28]. Topoisomerase II expression was also reduced by Pac/^212^Pb-trastuzumab (Untreated *vs* Pac/^212^Pb-trastuzumab, *p*<0.01; Pac/^212^Pb-trastuzumab *vs* Pac/^212^Pb-HuIgG, *p*<0.01), suggesting possible chromosome instability through perturbation of SAC proteins. Pac/^212^Pb-HuIgG elicited similar effects in the tumors, but, in this case, the levels of *BUBR1* and *TOPOISOMERASE II* were comparable to the untreated controls and much higher than that of the Pac/^212^Pb-trastuzumab treatment.

EMI1 (early mitotic inhibitor) suppresses APC/C activity during the cell cycle, and is believed to be required for proper mitotic entry. EMI1 depletion induces re-replication due to premature activation of APC/C that results in destabilization of GEMININ [29], [30]. The effect on EMI1 and GEMININ in tumors exposed to Pac/^212^Pb-trastuzumab was examined using immunoblot analysis. Pac/^212^Pb-trastuzumab reduced the expression level of EMI1 and GEMININ to a greater extent than paclitaxel alone (Figure 2B), implicating aberrant DNA re-replication which may result in DNA replication fork collision and double strand breaks.

## Discussion

The therapeutic potential of ^212^Pb-trastuzumab, α-emitting radioimmunotherapeutic, has been successfully demonstrated for the treatment of disseminated peritoneal disease in murine models [31]. Studies investigating the molecular basis of this efficacy have revealed that ^212^Pb-trastuzumab results in the induction of apoptosis, G2/M cell cycle arrest and blocks double strand DNA damage repair [32]. The molecular basis of this action is thought to be mediated through the p73/GADD45 signaling pathway via p38 kinase signaling [33]. Addition of paclitaxel to the treatment protocol resulted in greater therapeutic efficacy in the LS-174T i.p. tumor xenograft model [15]. The inclusion of paclitaxel in the regimen was found to increase mitotic catastrophe and apoptosis. Concomitantly, a redistribution of DNA content into the G2/M phase of the cell cycle, a decrease in the phosphorylation of histone H3, an increase in multi-micronuclei, and an increase in positively stained γH2AX foci, suggested possible effects on the mitotic spindle assembly checkpoint (SAC) by this combined modality therapy. Paclitaxel induces the arrest of spindle assembly checkpoint (SAC) through suppression of the spindle microtubule dynamics by binding to the β-subunit of tubulin and stabilizing microtubules. The resultant mitotic arrest rapidly triggers onset of the p53-independent apoptotic pathway [34]. To better understand the interplay between ^212^Pb-trastuzumab and paclitaxel that produces enhancement of the α-radiation cytotoxicity, gene expression profiling was performed with LS-147T i.p. tumor xenografts treated *in vivo* to identify affected genes.

Eighty-four genes were assessed using a real-time quantitative PCR (qRT-PCR) array. Thirty genes were identified (Tables 1-3) in the LS-174T tumors that were differentially expressed 24 h following the administration of paclitaxel followed the next day by ^212^Pb-trastuzumab treatment. Differential expression of eight genes had been previously identified following treatment with ^212^Pb-trastuzumab alone [33]. In each of the categories that were evaluated, apoptosis, cell cycle regulation and damaged DNA repair, more genes were affected by the combination of paclitaxel with ^212^Pb-trastuzumab. This is perhaps not surprising considering that paclitaxel was added to the treatment regimen to introduce other mechanisms of effecting or enhancing tumor cell killing. What is more striking when comparing the two studies is that more genes were down-regulated in their expression by the Pac/^212^Pb-trastuzumab, nine of which are involved in DNA repair. This suggests a more compromised cancer tissue in its effort to overcome the stress induced by the combined modality.

Eleven genes in the apoptosis panel demonstrated altered expression in the LS-174T tumors following treatment with Pac/^212^Pb-trastuzumab. Six *(CIDEA, GADD45α, GADD45γ, IP6K3, PCBP4* and *p73*) were found previously to be affected by ^212^Pb-trastuzumab [33]. More important, however, is that paclitaxel enhanced the expression of five of the genes, with two (*CIDEA* and *IP6K3*) having a 2.9- and 3.9-fold increase over the ^212^Pb-trastuzumab alone. Interestingly, even though the expression of *p73* was increased, the expression was lower in this study as compared to its expression following ^212^Pb-trastuzumab alone. Overall, the combined modality appears to have had the greatest effect on the expression of *CIDEA, GML* and *IP6K3*. These three are involved in apoptosis with *GML* also having a role in cell cycle regulation. In the presence of paclitaxel, *GML* is hypothesized to monitor the status of microtubules and transduces a signal to activate apoptosis, cooperating with or enhancing the effect of paclitaxel [35]. *GML* has also been implicated in the enhancement of G2/M arrest and apoptosis following α-irradiation [20]. However, an increase in the expression of this gene was not detectable following the exposure to α-radiation alone, and differential expression by paclitaxel treatment alone was not significant. Thus the 26.4-fold (*p*<0.0004) increased expression of *GML* by the combined Pac/^212^Pb-trastuzumab treatment may be a special response of the cells to the prior paclitaxel treatment. Interestingly, Pac/^212^Pb-HuIgG, the non-specific control also increased *GML* expression by 2.7-fold (*p*<0.0023) indicating that the α-radiation and paclitaxel together are important for *GML* expression. Paclitaxel can also result in stress-reaction-induced apoptosis through the up-regulation of the *GADD* gene family [36], [37]. *GADD45α* and *PPP1R15A* were both up-regulated following the Pac/^212^Pb-trastuzumab treatment. Again, the higher level of *GADD45* expression noted in the present study and the addition of *PPP1R15A* to the list of responsive genes attests to the increased stress on the LS-174T tumors by the combined modality. *RAD21* (Rad21 homolog) is a central component of the cohesin complex which consists of *RAD21, SMC1, SMC3*, and *SCC3*. *RAD21* expression confers poor prognosis and resistance to chemotherapy and knockdown of *RAD21* results in enhanced sensitivity to chemotherapeutic drugs [38]. In this study, *RAD21* expression was reduced to a greater extent by Pac/^212^Pb-trastuzumab treatment than Pac/^212^Pb-HuIgG or paclitaxel alone. These results suggest that ^212^Pb-trastuzumab and to a lesser extent, also ^212^Pb-HuIgG might increase cell sensitivity to therapy by up-regulating genes involved in apoptosis and down-regulating genes involved in desensitizing cells to chemotherapeutics. For α-particle irradiation, micronucleus induction has a biphasic phenomenon containing a low-dose hypersensitivity characteristic and its dose response could be well stimulated with a state vector model where radiation-induced bystander effects are involved. The increase in the micronucleus frequency in bystander cells provides evidence for indirect DNA damage signal or a bystander phenomenon released by irradiated cells. The non-specific effect in α-particle and bystander effects have been recognized in the past and occur as with β^−^-emitter therapy. Therefore, consideration toward greater studies of these specific responses must be given to these effects of radiation.

A total of eleven genes in the cell cycle category were found to be differentially regulated in the Pac/^212^Pb-trastuzumab treated xenografts, five of which were cross-overs into the apoptosis category. Four genes (*GADD45α, GTSE1, PCBP4* and *SESN1*) had been found to have an altered expression following treatment with ^212^Pb-trastuzumab alone [33]. The effect of paclitaxel alone was less pronounced. *BRCA1* was one of the genes down-regulated by Pac/^212^Pb-trastuzumab. A deficiency in BRCA1 causes abnormalities in the S-phase checkpoint, G2/M checkpoint, spindle checkpoint and centrosome duplication [22]. *CHK1* is required to delay entry of cells with damaged or unreplicated DNA into mitosis. CHK1 protects cells against spontaneous chromosome missegregation and is required to sustain anaphase delay when spindle function is disrupted by paclitaxel. The requirement of CHK1 for spindle checkpoints has been elucidated [39]. Spindle checkpoint failure in CHK1-deficient cells correlates with decreased AuroraB kinase activity, and impaired phosphorylation and kinetochore localization of BUBR1 [40].

DNA damage corrupts the integrity and translation of essential information in the genome. Two major strategies for repair are single strand break repair and double strand break repair (DSBR). The former includes nucleotide excision repair (NER), base excision repair (BER), mismatch repair (MMR), while the latter encompasses non-homologous end joining (NHEJ) and homologous recombination [41]. Eighteen genes involved in DNA repair were affected in the LS-174T tumor xenografts following the Pac/^212^Pb-trastuzumab treatment. These genes represented those involved in damaged DNA binding (DDB, 5 genes), NER (2 genes), BER (3 genes), MMR (2 genes), DSBR (3 genes) as well as 3 others related to DNA repair. In comparison to ^212^Pb-trastuzumab alone [33], the addition of paclitaxel to the treatment regimen not only resulted in an alteration in more genes involved in DNA repair, but also affected each of the major repair pathways. Interestingly, most of the genes involved in DNA single and double break repair, were differentially down-regulated, suggesting that Pac/^212^Pb-trastuzumab impairs both single and double strand break repair. Expression of several genes involved in DNA repair may be determinant of tumor sensitivity to the anti-mitotic chemotherapy. Severe DNA double-strand breaks are known to be caused by α-emitters that are also inefficiently repaired, leading to cell death [6], [32], [42]. A lack of specificity in this category of genes is worth noting. A comparison of the tumor response between the Pac/^212^Pb-trastuzumab and the Pac/^212^Pb-HuIgG groups gives the impression that there is little difference in the differential expression of most of the responding genes. However, there are differences between the two groups in the expression levels of the *ATRX*, *BTG2* and *XPC* genes. Loss of ATRX (alpha thalassemia/mental retardation syndrome X-linked) protein and mutations in the *ATRX* gene are associated with genome instability, defects in the G2/M checkpoint, and altered double strand break (DSB) repair in alternative lengthening of telomeres pathway. Recent developments suggest that ATRX plays a variety of key roles at tandem repeat sequences within the genome, including the deposition of a histone variant, prevention of replication fork stalling, and the suppression of a homologous recombination-based pathway of telomere maintenance [43]. *BTG2* (BTG family member 2) is induced through a p53 dependent mechanism and that expression of BTG2 promotes the repair of DSBs and reduces apoptosis by blocking the damage signal from p-ATM(S1981) to Chk2(T68)-p53(S20) via the activation of Mre11 and PRMT1 [44].

BRCA1 associates with tumor suppressor and DNA damage proteins to form a large complex, BRCA1-associated genome surveillance complex (BASC), which recognizes and repairs aberrant DNA structures. BASC contains BRCA1, ATM and BLM as well as four subprotein complexes; 1) RAD50-MRE11-NBN, 2) MSH2-MSH6, 3) MLH1-PMS2 and 4) RFCA. All of the BASC proteins can also form complexes independent of BRCA1. BASC is a dynamic structure in which multiple complexes assemble and disassemble at various sites of BRCA1 functions, DSB being one example [21]. The proteins all share the potential to act either as sensors of abnormal DNA structure or as effectors of repair. These properties of BASC lend a great deal of flexibility to the structure and allows for a rapid response for repair of DNA aberrations [21]. *MSH2* and *NBN* were two of the genes in the profile that were found to be differentially down-regulated by Pac/^212^Pb-trastuzumab along with *BRCA1*. Not only is the ability to bind damaged DNA hindered with the loss of *BRCA1* and *NBN*, but there is also the loss of one of the DNA repair mechanisms with lowered expression level of *MSH2*. Additionally, down-regulation of BRCA1 reduces the mitotic index and triggers premature CYCLIN B1 degradation and decrease in genes that are involved in the spindle checkpoint, including MAD2, which are key components that inhibit the anaphase-promoting complex [23]. Consistent with this notion, expressions of CYCLIN B1 and MAD2 that increased upon paclitaxel treatment alone were abrogated upon subsequent treatment with ^212^Pb-trastuzumab or ^212^Pb-HuIgG, suggesting the role of BRCA1 as a sensor of abnormal DNA structure or as effector of repair failed, resulting in chromosome instability following the α-radiation treatment. The effects of the decrease in BRCA1 is expected to be more pronounced for the Pac/^212^Pb-trastuzumab treatment compared with the Pac/^212^Pb-HuIgG treatment because of the greater reduction in BRCA1 expression for Pac/^212^Pb-trastuzumab than ^212^Pb-HuIgG.

*p73* is a member of the *p53* tumor suppressor gene family and induces cell cycle arrest and cell death in response to DNA damage. Loss of p73 can lead to mitotic arrest defects and p73 regulates the spindle checkpoint by modulating BUBR1 activity [24]. In this study, Pac/^212^Pb-trastuzumab treatment seemed to marginally increase the expression of *p73* at the gene level. However, expression of its downstream effectors, *NOXA* and *PUMA* at the transcriptional level was reduced. *p73* and *BRCA1* have also been shown to be transcriptional targets of E2F1. ChIP analysis revealed that the abundances of E2F1 on *p73* and *BRCA1* promoters were also down-regulated. These results were discordant. It would have been expected that increased expression of *p73* would result in an increased expression of its downstream effectors as well as an increase in the interaction with E2F1. These effects are most likely a result of the aberrant regulation, or lack thereof, of the BRCA1-associated target genes such as CHK1, E2F1, and p73 in response to Pac/^212^Pb-trastuzumab. It must be emphasized that the *in vivo* mechanisms invoked after the massive damage to DNA by the α-radiation are complex. Thus, we may not be able to explain all the results because of the myriad responses to the injury. However, the results obtained with the downstream effectors of *p73* indicate that the E2F1/p73 signaling pathway becomes defective after the combined paclitaxel and α-radiation treatment. p73 interacts with spindle assembly checkpoint (SAC) proteins and that loss of p73 causes mislocalization at the kinetochore and reduced kinase activity of BUBR1, leading to chromosome instability [24]. Studies from this laboratory recently demonstrated that paclitaxel potentiates ^212^Pb-trastuzumab induced cell killing by perturbing the mitotic spindle checkpoint, including BUBR1 [15]. Reduced BUBR1 activity results in the premature activation of the anaphase promoting complex/cyclosome (APC/C), which negatively regulates its substrates such as GEMININ [27]. In this study, α-radiation also suppressed EMI1 (early mitotic inhibitor), leading to unscheduled anaphase promoting complex/cyclosome (APC/C) activity as evidenced by down-regulation of GEMININ, an APC/C substrate, which plays redundant roles in preventing re-replication. The resulting aberrant DNA re-replication may result in DNA replication fork collision and double strand breaks. The presence of premature sister chromatid separation and chromosome breaks will compromise cell division and survival of the cells.

**Figure 3:**
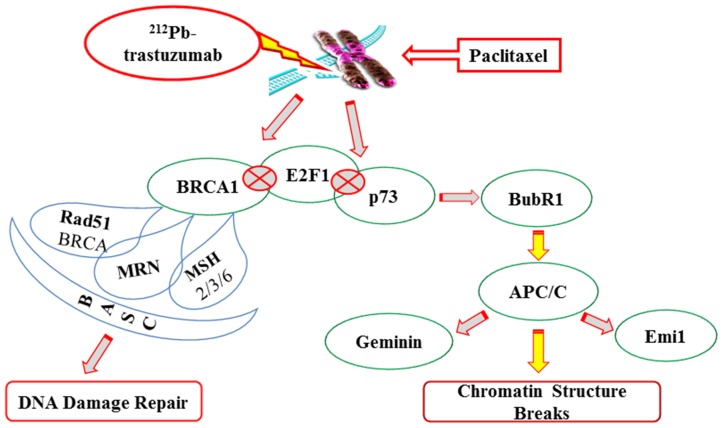
Proposed mode of action in the induction of chromosomal instability by Pac/^212^Pb-trastuzumab treatment. See text for details.

Clearly, the addition of paclitaxel resulted in a greater number of genes responding to the therapy especially those involved in DNA repair compared to ^212^Pb-trastuzumab treatment alone. The ^212^Pb-trastuzumab treatment is either potentiating the effect of paclitaxel, or, the paclitaxel is enhancing the effect of the ^212^Pb-trastuzumab. The findings suggest that perturbation of DNA damage repair and the mitotic checkpoint in tumors exposed to paclitaxel and ^212^Pb-trastuzumab may be responsible for the cell death. A fine cross-talk between DNA damage and the spindle damage response is evident (Figure 3). It must be noted that non-apoptotic death during mitotic catastrophe cannot be excluded as typically there is a mixture of apoptotic and non-apoptotic cell death during mitosis and after multinucleation. These studies represent a starting point for future investigations that will focus on the refinement of the identification of genes pivotal to the therapeutic response evoked by the combination of paclitaxel and targeted α-radiation using monoclonal antibodies such as trastuzumab. Analysis of later time points may reveal greater differences between specific and non-specifically delivered α-radiation.

The impact of the xenograft study results on understanding findings pertaining to the ongoing clinical trial at this time is limited since this is a Phase 1 trial that evaluates safety of ^212^Pb-labeled trastuzumab. The real impact is more directed towards future trials wherein our understanding of the results of these pre-clinical studies on targeted α-particle RIT might be applied to improve integration of this modality with standards of care chemotherapy, particularly so with respect to normal tissues toxicity as doses increase. Further elucidation of these mechanisms could aid in the development of more precise diagnostic and prognostic tools to promote clinical transition in the treatment of cancer.

## Supporting Information

Table S1 **Functional gene grouping.** Comparison of the relative expression of 84 DNA damage related genes involved in apoptosis (Figure 1), cell cycle (Figure 2), and DNA damage repair (Figure 3) was characterized with the human DNA damage signaling pathway PCR array.
(PPT)
