# Supplementary material for: Impact of α-Targeted Radiation Therapy on Gene Expression in a Pre-Clinical Model for Disseminated Peritoneal Disease when Combined with Paclitaxel
Source: PLoS One. 2014 Sep 30;9(9):e108511. doi: 10.1371/journal.pone.0108511 (PMC4182481; doi:10.1371/journal.pone.0108511)
Supplement: Table S1 — Functional gene grouping. Comparison of the relative expression of 84 DNA damage related genes involved in apoptosis (Figure 1), cell cycle (Figure 2), and DNA damage repair (Figure 3) was characterized with the human DNA damage signaling pathway PCR array. (PPT) [file pone.0108511.s001.ppt]

## Slide 1
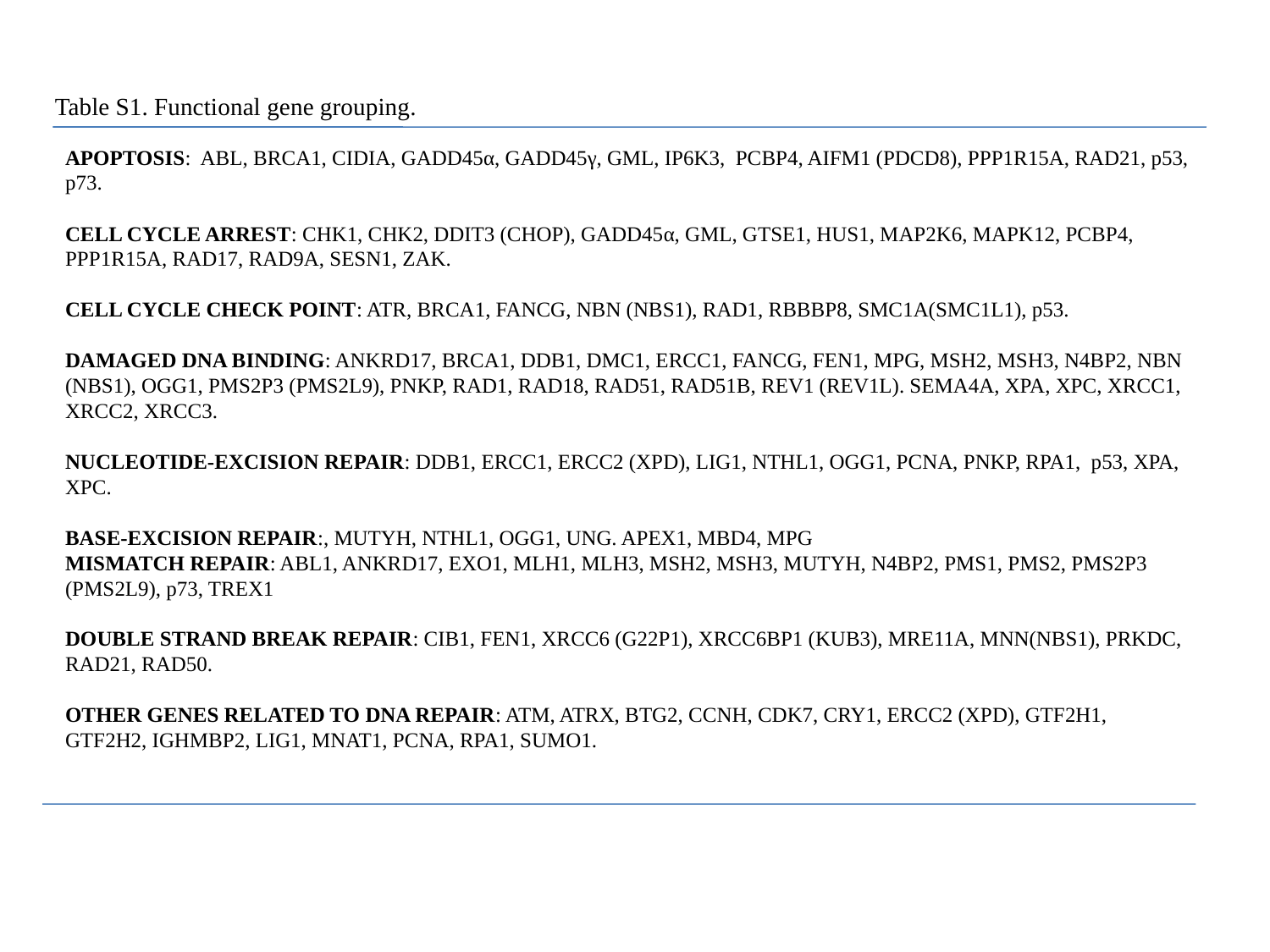

Table S1. Functional gene grouping.
APOPTOSIS: ABL, BRCA1, CIDIA, GADD45α, GADD45γ, GML, IP6K3, PCBP4, AIFM1 (PDCD8), PPP1R15A, RAD21, p53, p73.
CELL CYCLE ARREST: CHK1, CHK2, DDIT3 (CHOP), GADD45α, GML, GTSE1, HUS1, MAP2K6, MAPK12, PCBP4, PPP1R15A, RAD17, RAD9A, SESN1, ZAK.
CELL CYCLE CHECK POINT: ATR, BRCA1, FANCG, NBN (NBS1), RAD1, RBBBP8, SMC1A(SMC1L1), p53.
DAMAGED DNA BINDING: ANKRD17, BRCA1, DDB1, DMC1, ERCC1, FANCG, FEN1, MPG, MSH2, MSH3, N4BP2, NBN (NBS1), OGG1, PMS2P3 (PMS2L9), PNKP, RAD1, RAD18, RAD51, RAD51B, REV1 (REV1L). SEMA4A, XPA, XPC, XRCC1, XRCC2, XRCC3.
NUCLEOTIDE-EXCISION REPAIR: DDB1, ERCC1, ERCC2 (XPD), LIG1, NTHL1, OGG1, PCNA, PNKP, RPA1, p53, XPA, XPC.
BASE-EXCISION REPAIR:, MUTYH, NTHL1, OGG1, UNG. APEX1, MBD4, MPG
MISMATCH REPAIR: ABL1, ANKRD17, EXO1, MLH1, MLH3, MSH2, MSH3, MUTYH, N4BP2, PMS1, PMS2, PMS2P3 (PMS2L9), p73, TREX1
DOUBLE STRAND BREAK REPAIR: CIB1, FEN1, XRCC6 (G22P1), XRCC6BP1 (KUB3), MRE11A, MNN(NBS1), PRKDC, RAD21, RAD50.
OTHER GENES RELATED TO DNA REPAIR: ATM, ATRX, BTG2, CCNH, CDK7, CRY1, ERCC2 (XPD), GTF2H1, GTF2H2, IGHMBP2, LIG1, MNAT1, PCNA, RPA1, SUMO1.
